# Sleep Disturbances and Vitamin D in Celiac Patients Under a Gluten Free Diet: A Cross-Sectional Study

**DOI:** 10.3390/diseases14080279

**Published:** 2026-08-04

**Authors:** Giuseppe Losurdo, Francesco Squeo, Angela Marotti, Antonio Giangaspero, Enzo Ierardi, Mariabeatrice Principi

**Affiliations:** Section of Gastroenterology, Department of Precision and Regenerative Medicine and Jonian Area, University of Bari, 70124 Bari, Italy

**Keywords:** celiac disease, sleep, sleep disorder, vitamin D

## Abstract

**Background**: Celiac disease (CD) is an immune-mediated enteropathy associated with extra-intestinal manifestations. Sleep disorders may be frequent in CD patients. We aimed to investigate the quality of sleep in CD patients and to assess its relationship with clinical and biochemical parameters. **Methods**: We included patients with a proven diagnosis of CD under a gluten free diet at least twelve months prior to enrolment. All the participants completed the Sleep Scale from the Medical Outcomes Study (MOS sleep scale) and the Sleep Problems Index II was calculated. A high SLP9 score indicated a bad quality of sleep and values >20 revealed sleep disturbance. Moreover, blood samples were collected and demographic and clinical data were recorded from each subject. Linear logistic regression was used for uni- and multivariate analyses; the b coefficient and 95% confidence intervals (CI) were calculated. **Results**: We enrolled 53 patients, with the mean age being 42.75 ± 11.19 and 84.9% being females. The average hours of sleep per night were 6.47 ± 1.11. The mean SLP9 was 37.44 ± 20.65. Forty patients (75.5%) showed sleep disturbance, and 81.1% of patients had vitamin D insufficiency. According to univariate analysis, high SLP9 values (worse sleep) were inversely related to the number of sleep hours (b = −0.38, 95%CI: −11.8 to −2.2, *p* = 0.005) and to vitamin D levels (b = −0.57, 95%CI: −2.7 to −0.46, *p* = 0.009). In multivariate analysis only serum levels of vitamin D were inversely related to SLP9 (b = −0.47, 95%CI −12.8 to −0.43, *p* = 0.05). **Conclusions**: Low serum levels of vitamin D are independently associated with poor sleep quality in celiac patients. These findings need to be confirmed in future experimental and clinical studies.

## 1. Introduction

Celiac disease (CD) is an autoimmune enteropathy that, in genetically predisposed individuals, is triggered by gluten ingestion. Gluten is an umbrella term encompassing several peptides contained in wheat, barley and rye that in CD trigger an autoimmune response mediated by both antibodies and immune cells, leading to small intestinal mucosal atrophy and malabsorption [1].

Typical CD symptoms are linked to malabsorption and encompass diarrhea, abdominal pain, bloating and malnutrition. However, CD may disclose with some atypical symptoms such as anemia, hypertransaminasemia, and skin disorders and neurological symptoms such as dizziness, fatigue and a foggy mind, especially in adults [2,3].

Sleep disturbances are an overwhelming problem worldwide. An increasing interest in studying the quality of sleep in CD patients has been observed recently. These disorders are present in 10–22% of patients and may be the first clinical manifestations of CD in adults, along with potential intestinal symptoms for several years [4].

The increased risk of sleep disorders in patients with CD has been explained by some different mechanisms in previous studies. In patients who are not fully adherent to the gluten-free diet (GFD), villous atrophy could lead to malabsorption syndrome, with consequent nutritional deficiencies (e.g., vitamin B12, folic acid, and vitamin B6) [5]. However, nutritional deficiencies may also be a consequence of strict adherence to the GFD not supported by adequate supplementation. Indeed, many gluten-free grains are intrinsically poor in trace elements such as iron, thiamine, riboflavin, and niacin [6]. These trace elements participate in the synthesis of neurotransmitters such as tryptophan and melatonin, which are implicated in the regulation of various neurobiological mechanisms, including the regulation of the sleep–wake cycle. Tryptophan is an essential amino acid that plays a crucial role in the synthesis of serotonin and represents one of the most debated elements with respect to its possible role in the genesis of neuropsychiatric disorders in subjects affected by CD [7]. In this regard, several clinical proofs demonstrate the sleep-enhancing potential of vitamins, especially B vitamins, and vitamin D’s role in improving sleep quality in subjects with sleep disorders. As an essential co-factor for aromatic L-amino acid decarboxylase, vitamin B6 has a relevant function in improving sleep mainly by controlling the synthesis of melatonin. Furthermore, vitamin D has a central role in sleep regulation through genomic regulation of tryptophan hydroxylase 2 (TPH2) expression. Vitamin D deficiency slows the rate of slow-wave activity during restorative sleep, which can disrupt sleep homeostasis and circadian rhythm signaling [8]. Electroencephalogram studies in CD children demonstrated altered spindle activity with the presence of sleep disturbances [9]. In fact, in some studies it has emerged that sleep disorders are frequent in CD patients both at the time of diagnosis and during GFD adherence [10,11]. Nevertheless, in a study performed on a large cohort of American patients, a higher prevalence of insomnia did not emerge in celiac patients compared to a control group [12]. Most studies have been conducted on pediatric populations, but the results of these studies are discordant even in this case [13,14,15].

On these bases, the purpose of this study was to investigate the quality of sleep in CD patients and to assess the possible relationship between poor sleep quality and biochemical parameters.

## 2. Materials and Methods

### 2.1. Patients Selection

We planned a transversal, cross-sectional study. We recruited consecutive CD patients attending the Celiac Disease outpatient clinic of our Gastroenterology Unit in the period of October 2024–March 2025. The study was reviewed and approved by the Ethics Committee Board of University Hospital Policlinico of Bari (protocol no. 11907/11/02/2021) and was conducted in accordance with the Declaration of Helsinki. All patients gave written informed consent. The study was planned according to STROBE indications and the corresponding checklist is reported in Supplementary Material Table S1.

We selected patients over 18 years old who had a histological diagnosis of CD dating at least 12 months prior and are on a strict GFD for at least 12 months. Adherence to GFD was monitored by normalization of anti-transglutaminase antibodies. We excluded patients with poor GFD adherence and patients diagnosed with CD on a GFD for less than 12 consecutive months. Patients with comorbidities or conditions that could affect night sleep (e.g., psychiatric disorders, sleep apnea, pregnancy, or the presence of one or more dependent newborns) were excluded as well.

Demographic and medical history data were retrieved from each patient’s medical record. At each outpatient visit, the biochemical data obtained from blood tests performed close to the visit were recorded, including full blood count; iron status; folate, magnesium, vitamin B12, vitamin D, and albumin levels; and CD-specific autoantibodies. Each patient was asked if they had symptoms compatible with inadequately controlled CD (diarrhea, abdominal pain, and unwanted weight loss) at the time of the evaluation.

### 2.2. Quality of Sleep Evaluation

In order to evaluate the quality of sleep, during each outpatient visit, the Medical Outcomes Study (MOS) Sleep scale was administered to each patient. The MOS is a self-assessment questionnaire validated to study sleep disorders in the general adult population and in patients with chronic diseases of different origins [16]. The questionnaire consists of 12 items designed to investigate the number of hours of sleep per night and other parameters such as difficulty falling asleep, daytime sleepiness, snoring or the presence of elements that make nighttime rest discontinuous. To each of these questions, the patient could respond with a score between 1 and 6, where 1 indicated the continuous presence of the disorder, while 6 indicated the absence of the disorder. By performing a weighted average of the scores attributed to the answers relating to the items between the third and the twelfth, it was possible to calculate the Sleep Problem Index II (SLP9), which reflects a global evaluation index of the quality of nighttime rest. High SLP9 scores correspond to bad sleep quality, and scores ≥ 20 are indicative of significantly disturbed sleep.

The Sleep Somnolence Index (SLP3), an index that assesses daytime sleepiness, was also calculated by weighting the scores assigned to items 6, 9, and 11: high scores corresponded to great daytime sleepiness.

Furthermore, in order to evaluate the possible impact of psychophysical stress on sleep quality, the Perceived Stress Scale 4 (PSS-4) questionnaire was administered; it evaluates the levels of psychological stress perceived in the thirty days preceding the evaluation [17]. This questionnaire consists of 4 questions to which the patient can respond by assigning a score between 0 and 4, where 0 indicates the absence of stressful events taken into consideration while 4 indicates their continuous presence. The final score can vary from 0 to 16: higher PPS-4 scores are indicative of high levels of perceived stress.

### 2.3. Statistical Analysis

Continuous and dichotomous variables were presented as means ± standard deviations and proportions/percentages, respectively. Linear logistic regression was used for uni- and multivariate analyses, and the b coefficient and 95% confidence intervals (CI) were calculated. Variables with a *p* < 0.1 in the univariate analysis were included in the multivariate analysis. A *p*-value less than 0.05 (*p* < 0.05) was considered statistically significant.

Statistical analysis was performed using SPSS version 23 software (IBM Italy, Via Antonio Zanolini 36 AB, 40126 Bologna, Italy).

## 3. Results

We enrolled 53 patients with a mean age of 42.7 ± 11.2 years, with 84.9% being females. The mean age at diagnosis was 35.1 ± 11.3 years, and 73.6% (39/53) had villous atrophy at diagnosis. The most common symptoms were abdominal pain (54.7%), diarrhea (39.6%) and dyspepsia (47.1%). Seventeen patients (32.1%) had at least one further autoimmune disease, with the most common one being Hashimoto thyroiditis (n = 14, 26.4%).

Anemia was found in 27 patients (50.9%), and mean hemoglobin levels were 12.8 ± 1.5. Mean vitamin D levels were 22.2 ± 7.9 and insufficient levels were observed in 43 subjects (81.1%). Other demographic, clinical and laboratory features of the enrolled population are reported in Table 1.

The average hours of sleep per night were 6.47 ± 1.11 (Table 2). The mean SLP9 score was 37.44 ± 20.65. Therefore, according to the cut-off of 20, forty patients (75.5%) showed sleep disturbance. The average value of PSS-4 was 8.56 ± 2.23.

**Figure 1:**
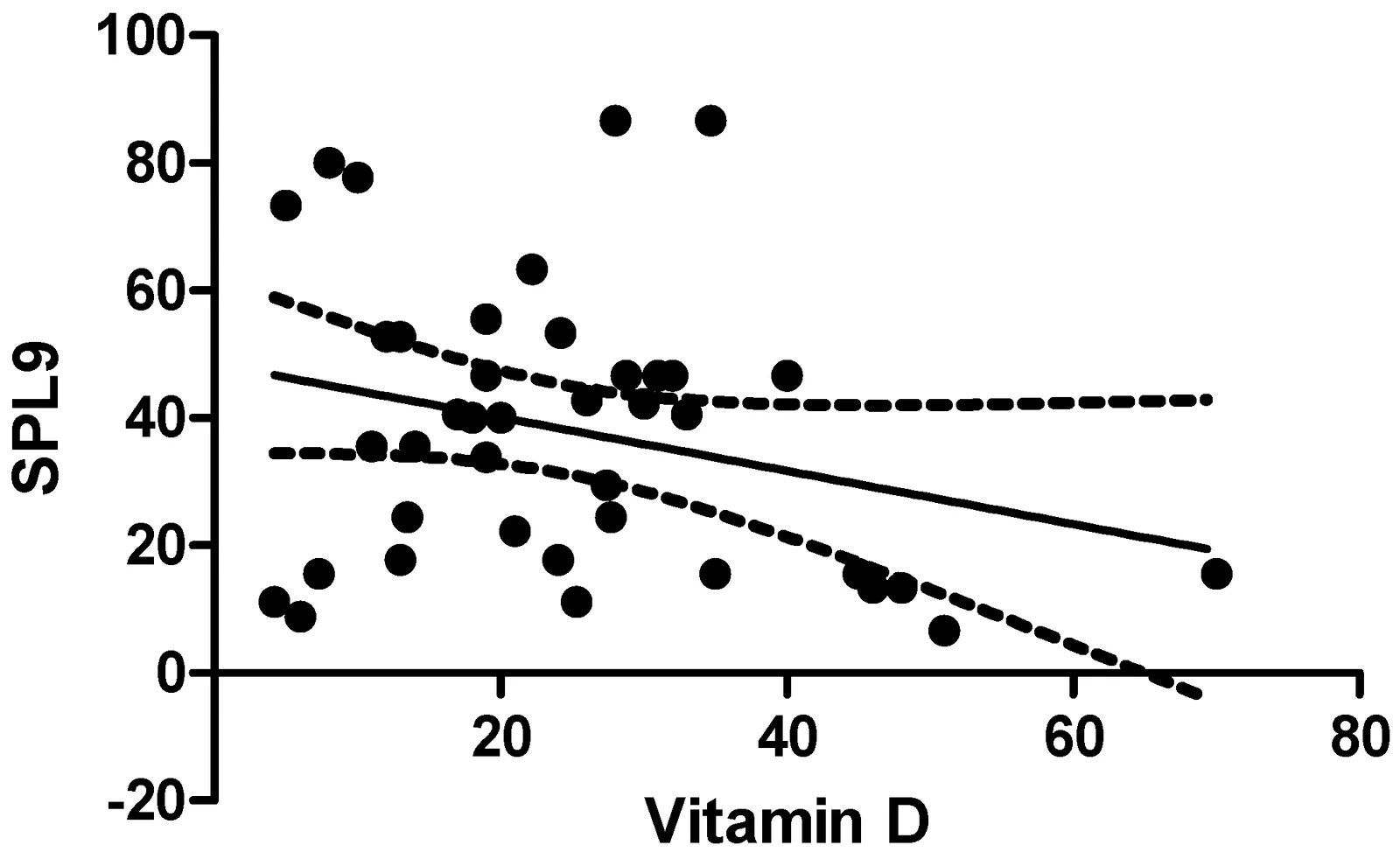
Scatterplot of linear regression between vitamin D and SPL9. The regression line is represented, along with its 95% confidence interval (dotted lines).

According to the univariate analysis, high SLP9 values (bad sleep) were inversely related to the number of hours of sleep (b = −0.38, 95% CI: −11.8 to −2.2, *p* = 0.005) and to vitamin D blood levels (b = −0.57, 95% CI: −2.7 to −0.46, *p* = 0.009). In Figure 1, the scatterplot describing the inverse relationship between vitamin D and SPL9 was reported. No correlation with other clinical or biochemical findings was found. In multivariate analysis only serum levels of vitamin D were inversely related to SLP9 (b = −0.47, 95% CI: −12.8 to −0.43, *p* = 0.05), as reported in Table 3.

No correlation between clinical and biochemical parameters and SLP3 and PSS4 was recorded.

## 4. Discussion

In the present study, sleep quality in a population with CD on a GFD was analyzed, assessing the associations among sleep, stress, and clinical and biochemical data. In recent years, growing interest in understanding the factors that can alter the quality of life in patients with various chronic diseases has prompted researchers to evaluate the impact of sleep in patients with gastrointestinal disorders such as CD.

In 2015, a case–control study conducted in Sweden on approximately 3000 celiac patients on a GFD and about 15,000 matched controls showed that celiac patients had a higher risk of insomnia and hypnotic drug consumption [10]. In another case–control study, Zingone et al. evaluated the quality of sleep in celiac patients at the time of diagnosis, in celiac patients on a GFD and in a control group using the Pittsburgh Sleep Quality Index (PSQI) [11]. The study showed that sleep disorders were more frequent in celiac patients than in the control group and that GFD did not lead to a statistically significant improvement in sleep quality. Furthermore, bad sleep quality was directly associated with the presence of stress, anxiety and depression and inversely associated with quality of life. On the other hand, another study found that a GFD significantly improved sleep scores in children with CD [18]. These arguable findings underline the complexity of the relationship between CD and sleep disorders. In this setting, our study confirmed that CD may show a very high rate of insufficient sleep quality (75.5%), even if no correlation has emerged between sleep quality and perceived stress levels.

Of interest, we found a strict correlation between vitamin D insufficiency and bad sleep quality in our CD population. The relationship between vitamin D and sleep quality is an intriguing finding. Clinical evidence is emerging about the potential beneficial effect of vitamins on sleep, especially B vitamins and vitamin D. For instance, vitamin D exerts its somnotrophic effects via genomic regulation of tryptophan hydroxylase 2 (TPH2) expression [19,20]. Vitamin D deficiency slows the rate of slow-wave activity during restorative sleep, which can alter sleep homeostasis and circadian rhythm signaling [21]. Recent meta-analyses have demonstrated an association between vitamin D deficiency and the presence of sleep disorders in the general population; furthermore, oral vitamin D supplementation appears to significantly improve sleep quality [22,23]. A three-way association between CD, vitamin D and sleep has been highlighted in a study showing a correlation between vitamin D levels and restless leg syndrome in CD subjects [24]. Vitamin D levels in CD are very frequently low at diagnosis and tend to increase and reach normal levels after GFD treatment [25]. Vitamin D levels may be often low in celiac disease patients primarily due to malabsorption. When untreated, consuming gluten damages the lining of the small intestine, causing villous atrophy. Additionally, since vitamin D is a fat-soluble vitamin, intestinal damage of CD may hamper absorption of fats and fat-soluble nutrients. Furthermore, low vitamin D levels are closely tied to low calcium levels. CD-related inability to absorb calcium can trigger secondary hyperparathyroidism, which further alters active vitamin D metabolism. Finally, many celiac patients may show dietary avoidance of some dairy products, leading to insufficient vitamin D intake [26]. Nevertheless, we found that most CD patients under a GFD showed insufficient levels. Unfortunately, we could not collect data about sunlight exposure, seasonal vitamin measurements and vitamin supplementation, and this is a study limitation that should be acknowledged.

It should be noted that the quality of sleep may depend on several other vitamins and minerals. Low selenium and magnesium levels were associated with sleep disorders in primary studies and meta-analyses [27,28]. The ability of selenium to counteract reactive oxygen species is the most plausible mechanism of action, as hypothesized by a recent trial [29]. Group B vitamins are crucial in mood regulation, and in a randomized clinical trial it was demonstrated that B1 and B2 supplementation improved the quality of sleep [30]. Vitamin B1 acts as a co-enzyme for adenosine triphosphate (ATP) production. While ATP is a universal intracellular energy source in multiple tissues, sleep is suggested to be necessary for replenishing ATP in the nervous system [31]. Finally, vitamin B6 is a relevant trace element as it is a co-enzyme that can participate in the synthesis of melatonin, which is generally considered an important hormone that affects sleep [32]. Since all these conditions are common in CD, clinical monitoring is necessary in these patients [33]. Unfortunately, we were not able to address whether selenium and most group B vitamins were involved in sleep disorders in our cohort of CD patients, since these elements are not routinely examined in our laboratory. This is a limitation that should be underlined.

Finally, it has been shown that the number of hours of sleep per night is an independent predictor of sleep quality in celiac patients. This is quite obvious, but a confirmation that short night sleep duration is associated with poor sleep quality and morning fatigue is described in the literature [34]. On the other hand, sleep quality depends not only on duration but also on the depth achieved during the first phase. Unfortunately, the nature of the MOS did not allow us to investigate sleep depth, and this is a limitation that should be acknowledged.

Among other results, our study did not reveal a correlation between SLP9 and the presence of autoimmune comorbidities, dermatitis herpetiformis, anemia, or first-degree family history. Several patients presented with symptoms such as diarrhea, dyspepsia, and abdominal pain at the time of evaluation; however, correlation analysis did not reveal an association with SLP9. It is therefore arguable to hypothesize that the presence of these symptoms, even at night, did not significantly affect sleep quality in the examined population. Even a relationship between SLP9 and stress, which is measured by PSS4, was not recorded.

Nevertheless, this study has some limitations due to the low number of enrolled patients, the lack of a control group, and experimental differences compared to other previous studies [11]; however, the main aim of our paper was to find possible factors associated with poor sleep in CD patients, rather than evaluating the risk of sleep disorders in CD patients compared to a healthy population. Also, we did not assess sleep quality at the time of diagnosis; therefore, a dynamic evaluation of whether a GFD in our cohort improved sleep was not possible. We did not collect data regarding menopausal state and related symptoms, which are related to worse quality of sleep [35], which is another limitation to mention. Additionally, vitamin D levels may strongly correlate with dietary habits and physical activity; unfortunately, we did not recall these data by interview or a dietary questionnaire, and this could be another limitation of our study. Considering some conflicting results, further studies conducted on a larger population are needed to validate these findings. Finally, the cross-sectional nature of our study only allowed us to establish a relationship between vitamin D and bad sleep, but we could not assess whether it is vitamin deficiency that cause sleep disorders or vice versa.

## 5. Conclusions

In conclusion, sleep disturbances have been shown to be common in celiac patients, even during GFD adherence. The presence of autoimmune comorbidities, first-degree family history, dermatitis herpetiformis, anemia, and gastrointestinal symptoms at the time of diagnosis is not associated with bad sleep quality. Among biochemical factors, normal serum vitamin D levels may have a protective effect on sleep quality. This result, however, requires more complex models requiring a sample size larger than that of the present study as well as a control group.

## Figures and Tables

**Table 1:** Main demographic, clinical and laboratory characteristics of the enrolled population.

Variable	Mean ± SD or n (%)
Age	42.7 ± 11.2
Sex M/F	8/45
Age at diagnosis	35.1 ± 11.3
Villous atrophy (Marsh 3) at diagnosis	39 (73.6%)
Symptoms	
Diarrhea Abdominal pain Dyspepsia Weight loss Anemia Depression Headache	21 (39.6%) 29 (54.7%) 25 (47.1%) 15 (28.3%) 27 (50.9%) 2 (3.8%) 2 (3.8)
Other autoimmune diseases	
Hashimoto thyroiditis Type 1 diabetes During dermatitis	17 (32.1%) 14 (26.4%) 1 (1.9%) 7 (13.2%)
Hemoglobin (g/dL, n.v. 12.5–16.5)	12.8 ± 1.5
Iron (µg/dL, n.v. 33–193)	61.9 ± 36.3
Ferritin (ng/mL, n.v. 13–150)	36.3 ± 49.2
Transferrin (mg/dL, n.v. 250–380)	268.1 ± 73.4
Albumin (g/dL, n.v. 3.5–5.5)	4.16 ± 0.32
Vitamin D, 25(OH) form (ng/mL) (n.v. > 20)	22.2 ± 7.9
Folate (ng/mL, n.v. 2.7–17)	5.3 ± 3.5
Vitamin B12 (pg/mL, n.v. 200–950)	356.4 ± 200.8
Magnesium (mg/dL, n.v. 1.7–2.2)	2.01 ± 0.18
Total cholesterol (mg/dL, n.v. < 200)	159.6 ± 36.8

**Table 2:** Factors related to sleep quantity and quality in the enrolled population.

Sleep Quality Indicator	Mean ± SD
Sleep hours	6.47 ± 1.11
SLP9	37.44 ± 20.65
SLP3	40.27 ± 22.78
PSS4	8.57 ± 2.22

**Table 3:** Univariate and multivariate analyses of factors correlated with sleep quality (SLP9 score).

	Univariate Analysis		Multivariate Analysis	
	b (95% CI)	*p*	b (95% CI)	*p*
**Age at diagnosis**	−0.75 (−0.65 to 0.37)	0.59		
**Anti-tTG at diagnosis**	−0.58 (−0.10 to 0.65)	0.68		
**Vitamin D**	−0.57(−2.7 to −0.46)	**0.009**	−0.47 (−12.8 to −0.43)	**0.05**
**Season of vitamin D testing (winter as reference)**	0.12 (−1.12 to 2.03)	0.64		
**Vitamin B12**	0.32 (−0.10 to 0.69)	0.13		
**Folate**	0.16 (−1.55 to 3.33)	0.46		
**Total cholesterol**	0.22 (−1.69–4.69)	0.65		
**Sleep hours**	−0.38 (−11.8 to −2.2)	**0.005**	−0.19 (−12.6 to 2.69)	0.40
**PSS4**	0.11 (−1.60 to 3.59)	0.44		

CI: confidence interval; tTG: tissue transglutaminase antibodies.

## Data Availability

The data presented in this study are available upon request from the corresponding author. The data are not publicly available due to ethical restrictions.

## Supplementary Materials

The following supporting information can be downloaded at https://www.mdpi.com/article/10.3390/diseases14080279/s1, Table S1: STROBE Statement—Checklist of items that should be included in reports of ***cross-sectional studies***.

## Abbreviations

The following abbreviations are used in this manuscript:

CD	Celiac disease
GFD	Gluten-free diet
PSQI	Pittsburgh Sleep Quality Index
MOS	Medical Outcomes Study
